# A distributed approach to the regulation of clinical AI

**DOI:** 10.1371/journal.pdig.0000040

**Published:** 2022-05-26

**Authors:** Trishan Panch, Erin Duralde, Heather Mattie, Gopal Kotecha, Leo Anthony Celi, Melanie Wright, Felix Greaves

**Affiliations:** 1 Division of Health Policy and Management, Harvard T.H. Chan School of Public Health, Harvard University, Boston, Massachusetts; 2 Wellframe Inc., Boston, Massachusetts; 3 Population Health Management, Mass General Brigham, Somerville, Massachusetts; 4 Department of Biostatistics, Harvard T.H. Chan School of Public Health, Harvard University, Boston, Massachusetts; 5 Institute for Medical Engineering and Science, Massachusetts Institute of Technology, Cambridge, Massachusetts; 6 Division of Pulmonary, Critical Care and Sleep Medicine, Beth Israel Deaconess Medical Center, Boston, Massachusetts; 7 College of Pharmacy, Idaho State University, Meridian, Idaho; 8 National Institute for Health and Care Excellence, London, United Kingdom; 9 Imperial College, London, United Kingdom; National Yang Ming Chiao Tung University, TAIWAN

## Abstract

Regulation is necessary to ensure the safety, efficacy and equitable impact of clinical artificial intelligence (AI). The number of applications of clinical AI is increasing, which, amplified by the need for adaptations to account for the heterogeneity of local health systems and inevitable data drift, creates a fundamental challenge for regulators. Our opinion is that, at scale, the incumbent model of centralized regulation of clinical AI will not ensure the safety, efficacy, and equity of implemented systems. We propose a hybrid model of regulation, where centralized regulation would only be required for applications of clinical AI where the inference is entirely automated without clinician review, have a high potential to negatively impact the health of patients and for algorithms that are to be applied at national scale by design. This amalgam of centralized and decentralized regulation we refer to as a distributed approach to the regulation of clinical AI and highlight the benefits as well as the pre-requisites and challenges involved.

The software and infrastructure needed to create AI has become cheaper and more ubiquitous leading to a rapid increase in the development of AI in the clinical context [1,2]. It is reasonable to expect this trend to continue and, in the future, for individual clinical organizations to routinely develop or adapt AI algorithms for their own purposes with their own data. However, clinical AI, like any technology in healthcare, is associated with risk including missed diagnosis, incorrect treatment, and exacerbation of inequity. The regressive impact of clinical AI on inequity was recently studied when a large insurer’s algorithm that generated clinical risk scores for patients based on their healthcare costs was analyzed. Because Black patients with similar disease severity to White patients typically access less care, and thus cost a payer less, the prediction model underestimated Black patients’ illness severity, resulting in less resources dedicated to Black patients compared with White patients for the same true illness severity [3]. While it has become more feasible to train such large models on large aggregations of data [4], there is limited evidence that these models generalize well in healthcare due to differences in the context the algorithm is developed in and the context the model in question is applied to; whether due to differences in clinical practice, differences in patient demographics, differences in healthcare utilization (as in the aforementioned example), or overfitting the model to the training data [5].

Regulation is necessary to ensure the safety, efficacy, and equitable impact of clinical AI. However, current regulatory approaches have been adapted from approaches designed to assess the safety and efficacy of drugs and conventional medical devices. Are these approaches sufficient for the challenges posed by new artificial intelligence technologies? The Food and Drug Administration (FDA) considers clinical AI as a software-based medical device. Typically, medical device approval is obtained via premarket clearance (510k), De Novo classification or premarket approval. Practically, this usually involves the approval of a “static” model after which reapplication must be carried out for any change in data, algorithm or intended use. Model performance must be demonstrated on an appropriately heterogeneous dataset, though this typically varies from application to application. More recently, the FDA has proposed a regulatory framework for *modifications* to AI, within the context of “Software as a Medical Device” (SaMD). This expands on the existing approach with new post-authorization considerations that are of greater importance for clinical AI [6]. Specifically, predetermined change control plans are recommended which place the onus on the *manufacturers* of algorithms to specify which parameters they intend to modify in future as well as the intended methodology to operationalize changes [7].

Our opinion is that, at scale, centralized regulation of clinical AI *only* is unlikely to adequately ensure the safety, efficacy, and equity of implemented systems. There are four specific factors that make centralized regulation at scale challenging:

First, the comparative ease of developing a new AI algorithm, compared to a new drug or conventional physical medical device, is expected to create a volume problem for existing regulators [8]. Fig 1 shows the number of AI/ML-enabled medical devices with FDA publicly available information by year from 2014–2020 [9]. Assuming a linear relationship between time and number of submissions, the projected load of submissions in 2025 would be 185 and in 2030 would be 274. Assuming that regulatory resources are as efficient in future and at greater scale this would represent a doubling of regulatory resources by 2025 and almost a tripling by 2030. We believe this volume of clinical AI is an underestimate and the trend of development will be non-linear as the technologies involved mature and cost of production falls as previously stated.

**Fig 1:**
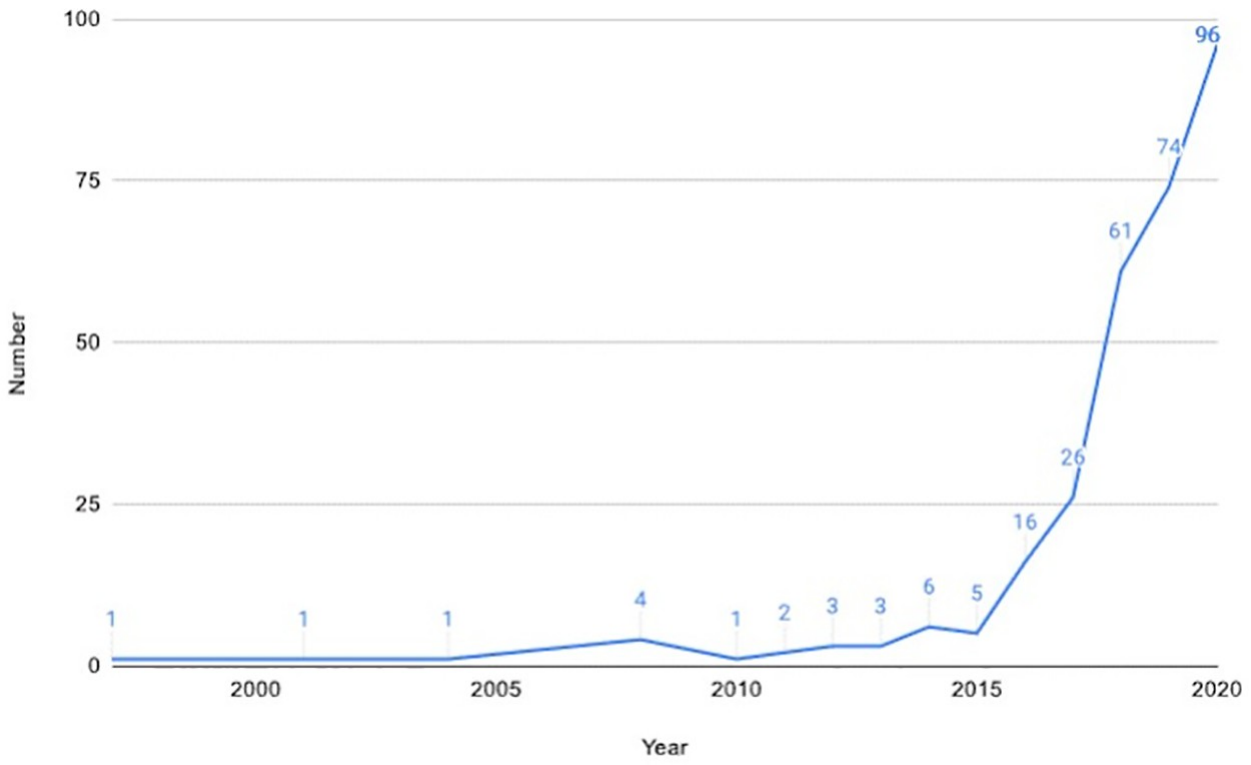
Number of AI/ML-enabled devices by year of FDA decision date. Data extracted from [9].

Second, AI technologies should necessarily change in response to changes in the underlying data [8]—compounding the aforementioned volume problem.

Third, many clinical algorithms are not equipped to determine causal relationships. Therefore, the reason for failure is not necessarily known. Since the inner workings are often “black boxes,” failure cannot always be predicted a priori, and given substantial.

heterogeneity in data and use cases, this makes centralized regulation alone especially challenging [1].

Fourth, a specific AI technology or device, regulated in isolation, cannot account for local socio-technical factors that ultimately determine the outcomes generated by technology in healthcare [10,11].

We believe that to address these challenges, it is necessary to supplement centralized regulation, derived from the approach to regulating and overseeing drugs and medical devices, with a decentralized approach for a technology that can, and arguably in many cases should be, created, evaluated, and deployed locally. Decentralized regulation is used elsewhere within a framework of explicit standards including in financial audit, e.g., the International Financial Reporting Standards (IFRS) [12]. We propose a hybrid model of regulation and oversight, building on the model of algorithmic stewardship proposed by Eaneff et al. [13] where decentralized regulation is the default for most applications of clinical AI going forward. Centralized regulation would only be required for the highest risk tasks—those for which inference is entirely automated without clinician review, have a high potential to negatively impact the health of patients, or that are to be applied at national scale by design, for example in national screening programs. This amalgam of centralized and decentralized regulation we refer to as a *distributed* approach to the regulation of clinical AI.

## Pre-requisites

We argue that while a distributed approach is desirable in the long run, it is not currently feasible. We employed an informal, iterative, consensus-building approach among the authors to identify five pre-requisite conditions and new institutional roles necessary for effective distributed regulation.

### The specialty of clinical AI

The safe, fair, and effective deployment of clinical AI will require a specially trained clinical workforce forming a new medical specialty of clinical AI that has ultimate accountability for its deployment. This has been described by Eaneff et al. as “algorithmic stewardship” [13]. Cosgriff et al. describe the development of this new specialty that would oversee the prospective evaluation, deployment, continuous monitoring and re-calibration of clinical AI [14]. This “Department of Clinical AI” would help regulate AI in the context of all the challenges above. Regulatory agencies have recently released guidance for Good Machine Learning Practice (GMLP) relevant to this specialization [15]. This differs from clinical informatics, a field dedicated to decision support rules without a focus on continuously revised modeling using machine learning. In addition, the department will oversee the training of clinicians and data scientists in this rapidly evolving field that encompasses topics including, but not limited to, human-computer interaction, decision support implementation science and algorithmic fairness. The establishment of a dedicated clinical AI department would need to be paired with a separate oversight role. In the same way that Institutional Review Boards (IRBs) are responsible for overseeing data access to balance the risks and benefits to human subjects in the development of algorithms for clinical research, there is a need for separate oversight of local algorithm adjustments and model revision to protect patient confidentiality, ensure safe model performance, and prevent adverse impact on disparities [16,17]. In a similar manner to how IRB approval is currently scope- and time-limited, local continuous quality improvement oversight would need to be maintained throughout the life cycle of the AI in question.

### An accountability framework

Price et al. describe the potential liability for clinicians using machine learning-based algorithms and note that there is, “essentially no case law on liability involving medical AI” [18]. In these cases, applying general legal principles suggests that when a clinician follows the recommendations of an AI system and deviates from the standard of care and the patient comes to harm, the clinician may face liability [18]. Until this issue is addressed by case law or statute it will not be possible for local algorithmic stewardship to be effective. Rather than follow directly the existing regulatory pathways for drug development, or software, we suggest that novel regulatory pathways for AI need to chart a new path that more closely parallels the regulation and revalidation of “encoded clinical knowledge;” the body of knowledge that any practicing clinician is assumed to have at any point in time. Ground level implementation of accountability guidelines needs to be part of the re-engineering of clinical pathways that occurs when AI is introduced. For high-impact scenarios (such as when the clinician deviates from both the standard of care and AI recommendation), the guidelines could include advice such as appropriate documentation, discussion with patients, and solicitation of a second opinion.

### Open data

Implementing AI in clinical practice requires a higher degree of validation than in other areas [19]. Ensuring the reproducibility of algorithmic performance on the data used for development requires the sharing of this data with independent researchers. In clinical verticals where large open access benchmark datasets are available, these serve as a natural foundation for algorithm development and validation [20]. However, few of these datasets exist and thus, in the majority of clinical domains, there needs to be disclosure of data in areas where data ownership and privacy considerations are contested [21]. In addition, where algorithms are to be applied locally on data other than the data they were trained on, a local benchmark data set is also required for local validation [22]. Improved data sharing would go some way towards managing Challenges 4 (generalizability) and 2 (continuous change). However, overcoming these barriers requires coordination at the level of the institutions or professional societies.

### AI registries

While the use of pharmaceuticals and medical devices is generally well coded in electronic health records, the use of emergent digital technologies and AI-based interventions is less well identified. We usually know precisely who has been exposed to a new drug or a device, but it is often difficult to tell who has had a ‘dose of AI’. The comparative ease of developing new AI algorithms brings with it new headaches for clinical coding and billing: a new taxonomy of CPT codes would need to be developed and adopted by frontline healthcare organizations—no small feat. A clear record of which patients have been exposed to specific AI technologies will be necessary to enable monitoring of outcomes, tracking of safety issues and billing. This is necessary given Challenges 1 (ease of new development) and 2 (continuous change).

### Public engagement

The COVID-19 pandemic laid bare race-based healthcare inequities, creating an imperative to not only include, but also focus on, marginalized populations. Interrogation of algorithms for bias has turned out to be a non-trivial task that is better performed by groups with representation of vulnerable patient populations. While it is possible to do this nationally, due to the heterogeneity of populations, it is likely to be more effective locally. Most recently, the FDA held a public workshop on transparency in AI in healthcare [23]. From this, and other research, there is emerging support of public priorities of transparency of: a) data representativeness in AI, b) evaluation of AI models for bias, c) privacy protections, d) potential risks and their mitigation, and e) accountability if errors or injustice does occur [24–32]. The FDA is currently supporting research and development efforts toward developing a form of labeling, similar to nutrition or drug facts labels, that directly addresses these transparency concerns [24, 33–36]. In addition to this, further participatory research is necessary to determine whether patients and clinicians would accept the distributed approach described herein as equivalent to centralized certification.

## Conclusions

The expansion of AI is a long-term secular trend in technology and safety and fairness as well as effectiveness are essential to develop the clinician and patient trust necessary to realize the benefits of these technologies in healthcare. The black box nature of Deep Learning, the difficulties in establishing causation, and the concerns regarding generalizability are known risks inherent in AI. Even with technical advances to address these issues, including model explainability [37–38] and synthetic datasets to reduce bias [39], risks remain. As such, the need for regulation to ensure the safety, efficacy and fairness of *clinical* AI is indisputable. Interrogation of algorithms for bias, post-deployment monitoring and algorithm update for data and calibration drift are important and resource-intensive tasks that are better performed by groups with appropriate diversity based on the context of the local health systems where such algorithms will be applied. As such, in the long run, we believe that the best approach to regulating clinical AI in practice is to not regulate it centrally, in most instances, but rather to delegate the regulation of clinical AI to local health systems.

This assertion is based on three beliefs. First, even with conservative projections regarding the growth of clinical AI, without a radical expansion in funding of the national organizations currently responsible for regulation, it will be impossible to address the future volume of regulation necessary. Second, that the correct paradigm for analyzing clinical AI should be is as encoded clinical knowledge, rather than considering it as a product such as a pharmaceutical or medical device. Within this paradigm, clinical AI—like clinical practice—requires an accountability framework, norms of practice and associated data assets to ensure that outcomes are achieved, patients protected, and equity improved [24]. The practice of medicine is not currently regulated in the same way as drugs or medical devices are, yet the current approach to regulation of clinical AI implies that when codified in machine learning algorithms it should be. Third, that it will be clinicians rather than manufacturers of algorithms who will, in the foreseeable future, be responsible for the implementation of clinical AI with patients. These clinicians will be faced with decisions about which specific patients might benefit from which specific algorithms and how those algorithms might exacerbate inequities across patient groups. Will broad certification from a national regulatory authority alone give these clinicians and the patients they serve sufficient peace of mind in making these decisions? We argue that the answer might well be no, as centralized regulation alone will not be able to address the reasons that algorithms might fail or increase disparities due to local context and practice patterns, thereby *inevitably* eroding trust.

Based on the confluence of these three beliefs, we argue that a distributed process involving a robust decentralized process as an adjunct to a centralized regulatory process is optimal. However, we accept that it is not currently possible. Such an approach requires the establishment of a specialty of clinical AI and an accountability framework, as well as the development of open data assets, AI registries, and a robust process for public engagement. It will also require a shift in regulatory mindset and an acceptance of changes in institutional responsibilities from existing regulatory organizations [40]. It is clear the incumbent approach will not scale and will necessarily erode patient and clinician trust as more algorithms are developed and both the regulatory backlog grows as do examples of algorithmic bias and the exacerbation of inequities. We appreciate that the distributed approach we have described here does not fundamentally change the regulatory burden inherent in ensuring the safety, equity, and fairness of clinical AI at scale, but rather it shifts the responsibility for ensuring safety, efficacy, and fairness to clinicians and healthcare organizations in partnership with patient representatives who, with adequate resources and collaboration, will be better placed to handle it. It is now up to existing regulatory organizations to propose an operational plan to realize a fairer and more effective *distributed* approach to the regulation of clinical AI.
